# Muscle Damage, Inflammation, and Muscular Performance following the Physical Ability Test in Professional Firefighters

**DOI:** 10.3390/sports11080144

**Published:** 2023-08-01

**Authors:** Matthew L. Sokoloski, Brandon R. Rigby, George A. King, Kyle D. Biggerstaff, Christopher J. Irvine, Andrew M. Bosak, Ryan A. Gordon, Emily L. Zumbro, Cayla E. Clark, Nicole L. Varone, Brett W. Crossland

**Affiliations:** 1 Exercise Physiology Laboratory, School of Health Promotion and Kinesiology, Texas Woman’s University, Denton, TX 76204, USA; msokoloski@twu.edu (M.L.S.); gking6@twu.edu (G.A.K.); kbiggerstaff@twu.edu (K.D.B.); rgordon4@twu.edu (R.A.G.); cclark33@twu.edu (C.E.C.); nvarone@twu.edu (N.L.V.); bcrossland@twu.edu (B.W.C.); 2 Department of Health and Human Performance, Rocky Mountain College, Billings, MT 59102, USA; christopher.irvine@rocky.edu; 3 School of Health Sciences: Allied Health Professions, Liberty University, Lynchburg, VA 24515, USA; ambosak@liberty.edu; 4 Department of Medicine, Division of Gerontology, Geriatrics, and Palliative Care, University of Alabama at Birmingham, Birmingham, AL 35209, USA; ezumbro@uabmc.edu

**Keywords:** first responder, tactical, exercise, cardiovascular disease, blood marker, strength, power, range-of-motion

## Abstract

Proper monitoring of fatigue and muscular damage may be used to decrease the high levels of cardiovascular disease, overuse musculoskeletal injuries, and workers compensation claims within the profession of firefighting. The purpose of this study was to examine muscle damage, muscular fatigue, and inflammation responses following a typical firefighting shift. Twenty-four professional firefighters completed two Physical Ability Tests to standardize the tasks typically performed in a day of work, and to elicit similar physiological responses. Participants were then monitored for 48 h. Prior to, and 48 h following the Physical Ability Tests, participants were evaluated for changes in strength, power, range-of-motion, as well as blood markers including myoglobin and c-reactive protein. Following the Physical Ability Tests, significant differences in myoglobin (*p* < 0.05), grip strength (*p* < 0.05), vertical jump (*p* < 0.05), and sit-and-reach (*p <* 0.05) were observed. No difference in c-reactive protein was observed (*p* > 0.05). After 24 hours following a shift, firefighters exhibited decreased strength, power, and range-of-motion. This may lead to decreases in performance and an increased risk of injury.

## 1. Introduction

Over 1 million people in the United States are professional or volunteer firefighters [1]. Whether performed professionally or on a voluntary basis, this is commonly classified as one of the most unpredictable, stressful, and dangerous occupations [2]. Every year, an average of 100 firefighters die while on duty [3]. Many of these deaths are attributed to complications from cardiovascular disease (CVD), with an increase in the incidence of these deaths within the last several decades [4,5]. Despite this, the overall risk of death attributed to CVD is similar to other occupations and the general population [6]. Strong associations between CVD and associated risk factors, including elevated blood pressure, high levels of triglycerides, low levels of high-density lipoproteins, glucose intolerance, and a sedentary lifestyle, exist in firefighters [7]. Other health concerns for CVD, such as poor dietary habits, chronically high levels of sympathetic stimulation, strenuous workloads, exposure to high temperatures, and poor sleep habits, are specific to the profession of firefighting [8].

The health and fitness levels of firefighters can be overlooked, particularly because many of these individuals are only subjected to fitness screenings once per year [9]. Furthermore, most departments throughout the United States do not mandate their first responders to exercise during their careers [9]. Professional firefighters that possess high levels of aerobic fitness, anaerobic capacity, muscular strength, and endurance have increased mobility, energy, and endurance [10]. This may allow these first responders to perform their occupational responsibilities in a safer and more efficient manner. Those with higher fitness levels may also be less likely to jeopardize the safety of their fellow firefighters and the public they serve while performing these duties. Despite this, mandating participation in a training program, and the tracking of health-related measures of physical fitness, are not mandatory for employment.

Tracking measures of fitness that impact performance is critical in the profession of firefighting. Immediately following strenuous physical activity, there is a significant drop in performance that lasts several days depending on the intensity, duration, and type of exercise [11]. The factors affecting this decrease in performance include myofibrillar disruption, swelling in the damaged area caused by an efflux of enzymatic activity, and the inflammatory process [11]. While the exact time to fully regain performance capabilities varies between individuals, muscle damage, resulting from a single bout of exercise, may elicit negative changes in range-of-motion [12], cognition [13], speed of muscular contraction [14], muscular strength, and VO_2max_ up to 96 h post-exercise [15]. An inability to fully recover from strenuous bouts of exercise can increase the risk of injury and further decrease optimal performance [16]. In addition to increased risk of injury, markers of muscle damage and inflammation are also elevated for 72 h post-exercise. Some of these markers have also been associated with an increased risk of myocardial infarction [17], metabolic syndrome, arthritis [18], and pulmonary disease [19]. These chronic diseases are among the most common causes of death and injury among firefighters.

Firefighting is a physically demanding occupation that requires firefighters to possess a high level of fitness and endurance. The physiological demands of firefighting can vary depending on the specific task and situation. These demands require that health-related measures of physical fitness, such as cardiovascular fitness, muscular strength, muscular endurance, and flexibility, be optimized [9]. To mitigate the risk of injury, and to assess performance, fire departments typically conduct an annual assessment to test the physical fitness of their city’s firefighters. Typically known as the Physical Ability Test (PAT), firefighters are required to perform tasks commonly performed while extinguishing a fire or during a search and rescue. Due to the PAT’s specificity to firefighting, this assessment is considered a reliable predictor of firefighting performance [20]. Some of these tasks, depending on department preferences, may include a dummy drag, repeated sledgehammer strikes, and ladder climb. Firefighters are instructed to complete these tasks as fast as possible and are given a score to determine their fitness level following the assessment.

Due to the high variability in call volume, firefighters may be asked to repeat maximal-effort activities, regardless if performance is hindered by activities completed in a previous call, or if the first responder is optimally recovered. These factors may allow for a higher risk of injury, which may be due to the incidence of muscle damage and inflammation. However, the relationship between these factors and recovery among firefighters is not well characterized. The combination of risk factors, lifestyle choices, and high physical workloads expected of professional firefighters greatly increases their risk of developing CVD or cardiovascular-related incidents [21]. The purpose of this study is to characterize markers of muscle damage, inflammation, and performance following the PAT in professional firefighters.

## 2. Materials and Methods

### 2.1. Participants

Twenty-four male professional firefighters, from the Dallas-Fort Worth area, were recruited using convenience sampling. Firefighters were recruited primarily from the fire stations of Addison, TX, and Carrollton, TX, USA, via word of mouth. All participants were recruited to include those who: (a) were employed as a full-time firefighter, and (b) identified as male. Firefighters were excluded from participation if they: (a) were unable to perform any task within the PAT, (b) were unable to attend or perform any task in the testing visits, (c) had a limiting musculoskeletal injury that precluded them from exercise, (d) reported the regular consumption of any medication that may influence inflammatory or CVD markers, or (e) were diagnosed with any cardiovascular, pulmonary, or metabolic disease. In addition, firefighters were excluded from participation in the study if they reported undergoing a stressful work shift or an atypical night of sleep that they felt would negatively impact their ability to participate. All participants were instructed not to perform any structured exercise 48 h prior to, and following, the PAT. Testing occurred on 16 October 2021 (22 °C, 84% humidity), 23 October 2021 (20 °C, 82% humidity), 13 November 2021 (10 °C, 70% humidity), and 28 November 2021 (10 °C, 80% humidity). All procedures were conducted in accordance with the Declaration of Helsinki and approved by the Institutional Review Board of Texas Woman’s University. Descriptive characteristics of the participants in this study (*n* = 24), as well as performance on the PAT (min), are outlined in Table 1.

### 2.2. Preliminary Testing Measures

All baseline testing occurred at the location of the PAT, 4798 Airport Parkway, Addison, TX, USA, starting between 0700 and 0900 h. Firefighters arrived in a fasted state (i.e., 10–12 h fast). During the preliminary visit, each participant provided written consent approved by the Institutional Review Board at Texas Woman’s University and completed a medical history questionnaire and the Physical Activity Readiness Questionnaire Plus (PAR-Q+). Preliminary testing was required due to the nature of scheduling at this fire department (24 h on and 48 h off). A double baseline approach ensured the participants in this study did not have elevated blood markers or decreased physical performance measures prior to participation.

### 2.3. Testing Measures

#### 2.3.1. Baseline Blood Draw

Following the completion of the necessary paperwork, participants sat upright for 5 min. A 10 mL blood sample was taken from the antecubital vein of the right arm. Blood samples were collected into BD Vacutainer K2 EDTA 10 mL tubes and immediately put on ice and transported to Texas Woman’s University (Denton, TX, USA) to be centrifuged at 3000 RPM for 10 min at 4 °C. Plasma samples were then aliquoted into cryotubes to be frozen at −80 °C for further analysis.

#### 2.3.2. Anthropometrics

Basic anthropometric data were also measured. Height (cm) was measured using a stadiometer (Perspective Enterprises Inc, Kalamazoo, MI, USA) and body mass was measured on a digital scale (Tanita LLC, Arlington Heights, IL, USA). Body mass index (BMI) was calculated from these measures. Following the anthropometrics, participants were given a standardized snack of 4 kcal/kg of body mass (Clif Bar & Company^®^, Emeryville, CA, USA; 250 kcal per 68 g serving; 18% fat, 68% carbohydrate, 14% protein) 15 min prior to beginning a dynamic warm-up.

#### 2.3.3. Performance Measures

##### Vertical Jump

Following the consumption of the snack and a 15 min rest period, participants underwent a dynamic warmup focusing on movements to increase functional mobility and decrease risk of injury for the participants (Table 2). Following the warm-up, participants were then instructed on how to perform a counter movement vertical jump. This test was performed using a Just Jump Mat (Perform Better^©^, Fredonia, NY, USA). The vertical jump test on the Just Jump Mat is a valid and reliable method to assess lower body power in adults [22]. Participants performed three vertical jumps, and the highest trial was used for data analysis

##### Hand Grip Strength

Hand grip strength was assessed using a Jamar hydraulic hand dynamometer (Sammons Preston, Bolingbrook, IL, USA). The measurement of grip strength via a hand grip dynamometer is a valid and reliable method to measure isometric strength in adults [23]. Firefighters sat with their shoulders adducted, elbows flexed 90°, and their forearms in neutral rotation. Firefighters were then asked to “squeeze as hard as possible” and given consistent verbal encouragement to squeeze “harder, harder, harder” during a 4.0 to 5.0 s effort. Two trials were allowed with each hand, with a 30 s rest between trials. The best scores of each hand were added together and averaged before being used for data analysis.

##### Flexibility

Trunk and hamstring flexibility was measured next using a sit-and-reach test (Novel Products^©^, Inc., Rockton, IL, USA). The sit-and-reach test is a valid and reliable method for assessing flexibility in the low back and hamstrings in adults [24]. For this test, firefighters removed their shoes and sat with their feet flat against the box. Keeping their legs extended and their hands overlapped, the participants reached forward slowly and pushed the pin on the box as far forward as possible. Participants were asked to hold the terminal position for 2 seconds and keep their knees extended throughout the test.

### 2.4. Physical Ability Test

Firefighters performed two rounds of identical PATs. The PAT consisted of 10 events: (a) 500 m row, (b) ladder climb, (c) hose drag, (d) stair climb with hose pack, (e) hose pull, (f) crawl, (g) stair descent, (h) Kaiser machine sledgehammer strikes, (i) hose couple, and (j) dummy drag. Following the first PAT, firefighters immediately performed the identical PAT again with no rest period separating the tests. All firefighters donned typical gear, including a 22 kg air tank strapped to their back, while performing both PATs. A detailed description of each test can be found in Table 3.

### 2.5. Post-Testing Procedures

**Figure 1:**
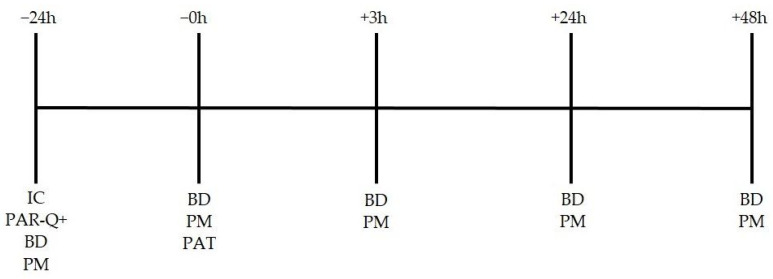
Study timeline. IC = informed consent; PAR-Q+ = Performance Activity Readiness Questionnaire; BD = blood draw; PM = performance measures; PAT = physical abilities test.

Following the PAT, firefighters immediately took off all firefighter equipment and rested. Baseline measures, including a blood draw, vertical jump, hand grip, and a sit-and-reach test, were administered 3 h after completion of the PAT. Firefighters were then sent home and returned to complete the same procedures 24 h and 48 h post-PAT at their respective fire station. A timeline of events can be seen in Figure 1.

### 2.6. Blood Analyses

All blood samples were obtained in the morning (starting between 0700 and 0900 h), with the exception of the first blood draw post-PAT (i.e., 3 h post-PAT), in a fasted state (i.e., 10–12 h) at the Addison fire station (Addison, TX, USA). Concentrations of c-reactive protein (CRP) and myoglobin (Mb) were analyzed with the Luminex MagPix℗ using a custom bead panel kit (EMD Millipore Corporation, Bellerica, MA, USA). Researchers followed the manufacturer’s instructions for all procedures, and all measures were performed in duplicate. Measured serum values found within expected normal physiological reference interval were recorded as the average of the duplicate results. Values that fell outside of the expected physiological reference interval were removed as outliers and the single measure within the range was recorded.

### 2.7. Statistical Analyses

An a prior power analysis (G*power 3.1.9.2, Dusseldorf, Germany) was conducted to determine the minimum sample size required to find statistical significance. With a desired power level of 0.80, an alpha (α) level set at 0.05, and a moderate effect size of 0.25 (f), it was determined that 24 participants would be required. The data were normally distributed, and a repeated measures analysis-of-variance (RM ANOVA) was performed to determine differences for all variables between pre- and post-PAT time points. Sidak post hoc testing was used to determine differences between time points when a main effect was observed. Significance in this study was set at *p* < 0.05. All statistical analyses were performed using SPSS statistical software (IBM SPSS Statistics v.28, Armonk, NY, USA).

## 3. Results

### 3.1. Participants

Twenty-six professional male firefighters were initially recruited. Two participants were dropped from data collection due to scheduling conflicts. Twenty-four firefighters completed all procedures. Inflammatory and muscle damage measures were not obtained on some participants due to error during blood marker analysis in the pre- and post-PAT periods.

### 3.2. Muscle Damage and Inflammatory Response

#### 3.2.1. Myoglobin Concentrations

**Figure 2:**
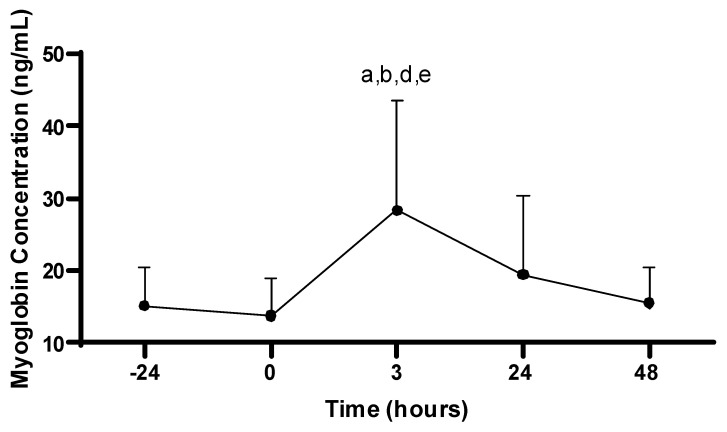
Myoglobin concentrations. Values are presented as mean ± SD; −24 = 24 h prior to Physical Ability Test (PAT); 0 = Immediately following PAT; 3 = 3 h post-PAT; 24 = 24 h post-PAT; 48 = 48 h post-PAT; a = significantly greater compared to −24 h (*p* = 0.005); b = significantly greater compared to 0 (*p* < 0.001); d = significantly greater compared to 24 h (*p* = 0.042); e = significantly greater compared to 48 h (*p* = 0.003). *n* = 21.

There was a main effect for Mb concentration across time (−24, 0, 3, 24, 48 h; *p* < 0.001). The Mb concentration at 3 h post-PAT (38.4 ± 15.2 ng/mL) was increased compared to all time points: −24 (15.2 ± 5.3 ng/mL; *p* = 0.005), 0 (13.8 ± 5.1 ng/mL; *p* < 0.001), 24 (19.5 ± 10.9 ng/mL; *p* = 0.042), and 48 h (15.6 ± 5.0 ng/mL; *p* = 0.003). No differences in Mb were observed between any other time points (*p* > 0.05; see Figure 2).

#### 3.2.2. C-Reactive Protein Concentrations

**Figure 3:**
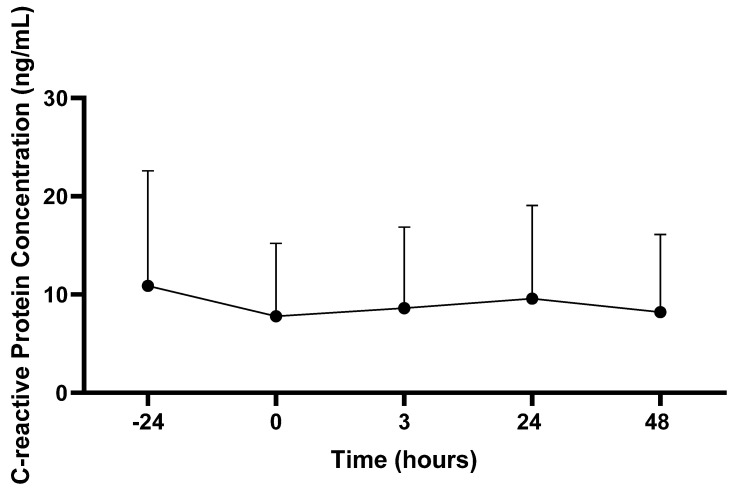
C-reactive protein concentrations. Values are presented as mean ± SD; −24 = 24 h prior to Physical Ability Test (PAT); 0 = Immediately following PAT; 3 = 3 h post-PAT; 24 = 24 h post-PAT; 48 = 48 h post-PAT. *n* = 22.

There was no main effect for CRP concentration (ug/mL) across the time (*p* > 0.05; see Figure 3).

### 3.3. Performance Measures

#### 3.3.1. Hand Grip Strength

**Figure 4:**
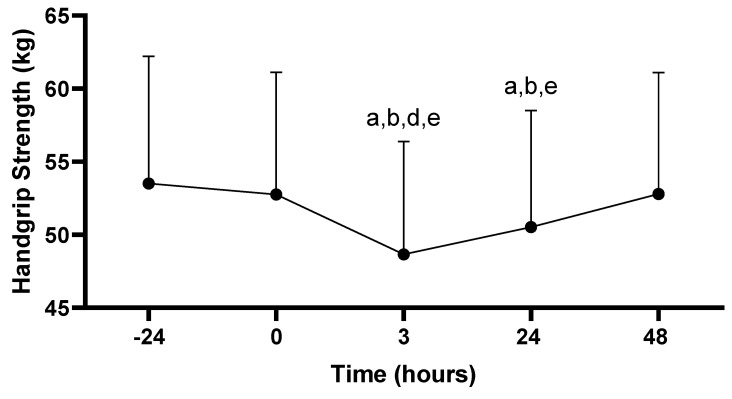
Hand grip strength scores. Values are presented as mean ± SD; −24 = 24 h prior to Physical Ability Test (PAT); 0 = Immediately following PAT; 3 = 3 h post-PAT; 24 = 24 h post-PAT; 48 = 48 h post-PAT; a = significantly lower compared to −24 h (*p* < 0.001); b = significantly lower compared to 0 (*p* < 0.05); d = significantly lower compared to 24 h (*p* = 0.001); e = significantly lower compared to 48 h (*p* < 0.001). *n* = 24.

There was a main effect for hand grip strength across time (−24, 0, 3, 24, 48 h; *p* = 0.007). Hand grip performance at 3 h post-PAT (48.6 ± 7.7 kg) was decreased compared to all time points: −24 (53.5 ± 8.7 kg; *p* < 0.001), 0 (52.8 ± 8.4 kg; *p* < 0.001), 24 (50.5 ± 8.0 kg; *p* = 0.001), and 48 h (52.8 ± 8.3 kg; *p* < 0.001). In addition, hand grip performance at 24 h post-PAT was decreased compared to −24 (*p* < 0.001), 0 (*p* = 0.004) and 48 h (*p* < 0.001). No other differences in hand grip were observed (*p* > 0.05; see Figure 4).

#### 3.3.2. Vertical Jump

**Figure 5:**
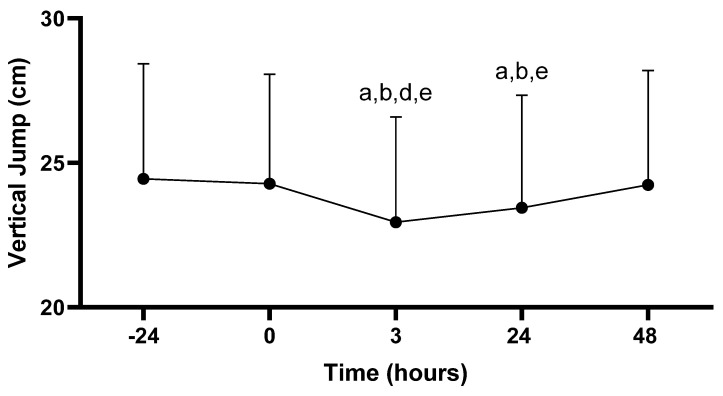
Vertical jump scores. Values are presented as mean ± SD; −24 = 24 h prior to Physical Ability Test (PAT); 0 = Immediately following PAT; 3 = 3 h post-PAT; 24 = 24 h post-PAT; 48 = 48 h post-PAT; a = significantly lower compared to −24 h (*p* < 0.05); b = significantly lower compared to 0 (*p* < 0.05); d = significantly lower compared to 24 h (*p* = 0.020); e = significantly lower compared to 48 h (*p* < 0.001). *n* = 24.

There was a main effect for vertical jump across time (−24, 0, 3, 24, 48 h; *p* < 0.001). Vertical jump performance at 3 h post-PAT (58.2 ± 9.1 cm) was decreased compared to all time points: −24 (62.0 ± 10.2 cm; *p* < 0.001), 0 (61.7 ± 9.7 cm; *p* < 0.001), 24 (59.4 ± 9.9 cm; *p* = 0.020), and 48 h (61.5 ± 10.2 cm; *p* < 0.001). In addition, vertical jump performance at 24 h post-PAT was decreased compared to −24 (*p* = 0.002), 0 (*p* = 0.005), and 48 h (*p* < 0.001). No other differences in vertical jump scores were observed (*p* > 0.05; see Figure 5).

#### 3.3.3. Flexibility

**Figure 6:**
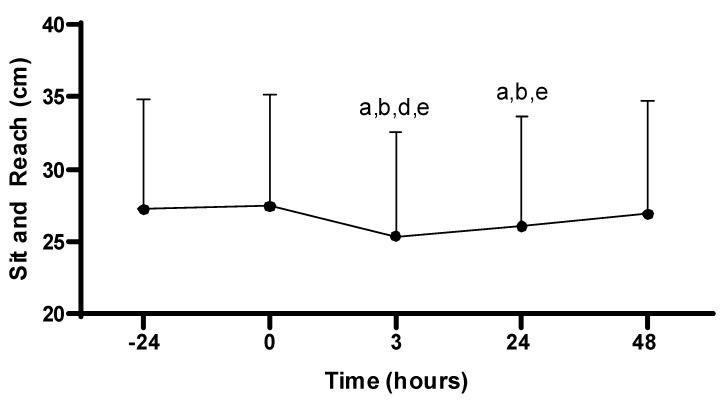
Sit-and-reach scores. Values are presented as mean ± SD; −24 = 24 h prior to Physical Ability Test (PAT); 0 = Immediately following PAT; 3 = 3 h post-PAT; 24 = 24 h post-PAT; 48 = 48 h post-PAT; a = significantly lower compared to −24 h (*p* < 0.001); b = significantly lower compared to 0 (*p* < 0.001); d = significantly lower compared to 24 h (*p* = 0.029); e = significantly lower compared to 48 h (*p* < 0.05). *n* = 24.

There was a main effect for the sit-and-reach across time (−24, 0, 3, 24, 48 h; *p* < 0.001). Sit-and-reach performance at 3 h post-PAT (25.4 ± 7.2 cm) was decreased compared to all time points: −24 (27.2 ± 7.6 cm; *p* < 0.001), 0 (27.4 ± 7.7 cm; *p* < 0.001), 24 (26.1 ± 7.6 cm; *p* = 0.029), and 48 h (26.9 ± 7.8 cm; *p* < 0.001). In addition, sit-and-reach performance at 24 h post-PAT was decreased compared to −24 (*p* < 0.001), 0 (*p* < 0.001), and 48 h (*p* = 0.013). No other differences in sit-and-reach were observed (*p* > 0.05; see Figure 6).

## 4. Discussion

This study aimed to characterize how systemic markers of muscle damage and inflammation, as well as markers associated with muscular performance, are affected following the PAT in professional firefighters. In this study, an increase in Mb concentrations was observed 3 hours following the PAT in firefighters. In addition, hand grip strength, vertical jump, and sit-and-reach performance was impaired for up to 24 h following the PAT. Within this occupational population, it is necessary to explore physiological responses to performing firefighter duties, while also trying to gain a greater understanding of how long it takes firefighters to fully recover from their occupational tasks. This may identify time periods as well as performance variables that are negatively affected by task performance, which may result in firefighters performing their job at a sub-optimal level. This information may then be translated to determining a firefighter’s level of fitness that prevents the onset of occupation-related CVD while also allowing them to perform their job at a high level.

### 4.1. Muscle Damage and Inflammation

Myoglobin, a commonly assessed marker of muscle damage (i.e., a relatively common phenomenon that occurs in response to intense mechanical load and force requirements [25,26,27]), was significantly increased 3 h following the PAT in this study. Concentrations gradually returned to basal levels 48 h post-PAT. In addition, CRP, a marker representative of localized and systemic inflammation, was assessed. There were no differences in CRP prior to, or following, the PAT. Collectively, it appears the PAT was strenuous enough to promote damage of muscle tissue in the early periods following the PAT, as evidenced by an increase in Mb within the circulation, though this was accompanied by no significant changes in CRP. The absence of a cytokine response in this study may be due to the intensity, duration, and acute nature of the PAT. Long-term structured exercise performed at higher intensities and over prolonged durations per session is more likely to elicit a cytokine response [28,29,30,31].

The observed increase in Mb following strenuous exercise has been documented in previous studies. Nybo et al. investigated markers of muscle damage and performance, including Mb, in semiprofessional soccer players in response to matches performed in both neutral and hot environments. The authors reported elevations in Mb immediately following both matches, with environmental temperature having no effect on Mb [32]. Also, Mb returned to basal levels at both 24 and 48 h post-exercise [32], similar to results reported in the current study. Ascensão et al. explored markers of oxidative stress and muscle damage in soccer players prior to, and during, a soccer match. The authors reported increases in Mb 30 min following the match, with Mb returning to pre-match levels 24 h post-match [33], which follows the results reported in the current study. Neubauer et al. assessed markers of inflammation and muscular stress in triathlon athletes following an Ironman competition. Myoglobin was significantly elevated immediately post-race compared to pre-race values, and Mb remained elevated up to 19 days post-exercise [34]. Similar to results in the current study, Neubauer et al. reported Mb concentrations peaked immediately after the competition. However, in contrast to the results of the current study, elevations in Mb were observed over a longer period of time following exercise, which can be attributed to the significant muscular and metabolic requirements of the Ironman competition [34]. Therefore, it appears Mb concentrations peak within a few hours following cessation of strenuous activity, and typically return to basal levels within 24 h post-exercise [35].

Despite changes in Mb within this study, no significant changes in CRP were observed in response to the PAT. As both Mb and CRP are associated with localized and systemic inflammation, it was expected that changes would occur with both markers in response to the PAT [36]. According to results in the current study, CRP concentrations remained relatively unchanged prior to, and following, the PAT, indicating little to no effect on CRP in response to the PAT.

Results for CRP in the current study are similar to observations from previous investigations. Specifically, no changes in CRP in response to high-intensity endurance events (e.g., 10 km, marathon) [37] and high-intensity resistance training [38] have been reported. Despite this, several studies have reported significant changes in CRP in response to exercise. Specifically, increases in CRP concentrations in response to ultra-endurance exercise [28,29] and high-intensity resistance training [30,31] have been documented, with these increases in CRP lasting 24 or more hours. The uniqueness of the PAT, in addition to differences in the methodology employed in the current study compared to previous studies, creates challenges when generalizing our results.

### 4.2. Muscular Performance

In this study, hand grip strength, vertical jump, and sit-and-reach performance were assessed before and after the PAT. Higher levels of hand grip strength are associated with improved time to completion for standardized tests for firefighters, including the hose drag and the stair climb with a high-rise pack [39]. The sit-and-reach test was performed in this study due to the need to assess lower body flexibility (including hamstring flexibility) among the participants. It is well established that poor hamstring range-of-motion is correlated with low back pain, and low back pain is a leading cause of disability in firefighters [40]. Reductions in all three performance variables were documented at 3 and 24 h following the PAT. This was expected, as the time period immediately following intense, or long-duration exercise is typically characterized by significant reductions in exercise performance that can persist for several days [11]. In addition, hand grip strength and vertical jump performance were significantly decreased at 3 hours post-PAT when compared to 24 h post-PAT, which indicates participant performance was negatively impacted at a higher magnitude in the hours immediately following the PAT compared to 24 h after the PAT was completed. The performance measures were not correlated to the markers of muscle damage or inflammation assessed in this study. It is possible that the PAT protocol used was not strenuous enough to elicit a cytokine response due to the lack of eccentric loading in the PAT [41]. This may be due to a lack of eccentric loading in our firefighter specific protocol.

It is well established that both aerobic and resistance training can improve hand grip strength [42], sit-and-reach [43], and vertical jump performance [44]. However, less is known regarding the effects of a single bout of exercise with these performance variables. García-Pinillos et al. explored the effects of acute interval training on countermovement jump performance and hand grip strength in endurance athletes. Participants performed twelve 400 m runs, with countermovement jump performance and hand grip strength measured after every three runs. The authors reported consistent improvements in both variables throughout the testing duration in comparison to baseline scores. Importantly, similar improvements were observed at the conclusion of the last 400 m run; thus, it appears participants were able to minimize the accumulating fatiguing effects of these 400 m runs on both their countermovement jump and hand grip strength [45]. The discrepancy between these results when compared to the results in the current study is likely due to differences in the type of tasks performed and participant exercise and training history.

In another study, the effects of repeated 20 min work bouts were investigated on a variety of performance variables in Australian firefighters [46]. Participants completed simulations consisting of two, 20 min search and rescue tasks in an environmentally controlled heat chamber at approximately 105 °F. Performance variables were measured at various points prior to, and following, each 20 min simulation. Similar to results in the current study, the authors reported that hand grip strength was significantly decreased after the second 20 min simulation in comparison to the first simulation [46]. Muscular strength may therefore be reduced in firefighters following intensive occupational-related tasks.

Michaelides et al. assessed the relationship between fitness measures and occupational task performance, including a stair climb, hose pull, and mannequin rescue drag in firefighters. The authors found several fitness variables predictive of performance with these tasks, including hand grip strength. However, vertical jump and sit-and-reach performance was not found to be predictive of ability test performance [20].

The results obtained for hand grip strength, vertical jump, and sit-and-reach performance in this study are similar to those reported in previous studies in which professional firefighters were participants. Several important concepts begin to emerge when considering our results, as well as those from previous studies exploring this topic. First, it is apparent that hand grip strength performance is sensitive to strenuous exercise, particularly during repetitive activities commonly performed by firefighters while on duty [45]. Second, vertical jump performance, an assessment of anaerobic power production, appears to be negatively affected in response to similar activities; however, improvements were reported in previous studies [45]. Third, the effects of acute exercise on sit-and-reach performance, particularly within the firefighter setting, are not well characterized in the literature. Despite this, it is well established that range-of-motion is typically reduced following strenuous exercise or physical activity [12], which is similar to our observations in this study. Finally, hand grip strength predicts occupational task performance in firefighters, while vertical jump and sit-and-reach performance do not [20]. Thus, it appears the measurement of hand grip strength may be of great significance within the firefighter population with regard to the prediction of task-specific performance.

### 4.3. Limitations

A central limitation to this study is the use of the PAT as a reflection of occupational tasks that a firefighter may experience. Though the PAT may be a reliable determinant of firefighting performance and task requirements [20], firefighters are frequently subjected to unpredictable and prolonged occupation-specific responsibilities and hazards, making it difficult to fully replicate the tasks these individuals may face during a shift. Additionally, the PAT was chosen as the form of exhaustive exercise in this study and does not follow traditional aerobic or resistance exercise protocols. Taking into consideration the structure and predictability of the PAT in comparison to more variable firefighter tasks (e.g., responding to a fire or emergency, multiple tasks in a shift, not aware of the task ahead of time), it is possible that the magnitude of muscle damage and inflammation may be greater during real-time firefighter tasks. Though challenging from a feasibility perspective, it would be beneficial to observe these markers of muscle damage and inflammation in response to real-time firefighter tasks. Muscle damage was not directly assessed in this study, though the markers used in this study (i.e., Mb, CRP) reflect muscle damage. Another limitation is that maximum exercise effort and maximal oxygen consumption (VO_2max_) were not directly measured. As such, exercise intensities, such as those experienced during the PAT, in terms of a percentage of HR_max_ or VO_2max_ or expressed as a percentage of HR or VO_2_ reserve are not reported. Additionally, in this study, we did not collect information on participants’ exercise history or years working as a firefighter. As both factors can influence performance on tasks such as the PAT, it is possible these factors could have influenced outcomes in this study. This should be taken into consideration when examining the results of this study.

## 5. Conclusions

In this study, an increase in Mb concentrations was observed 3 hours following the PAT in firefighters. In addition, hand grip strength, vertical jump, and sit-and-reach performance were impaired for up to 24 h following the PAT. These results indicate muscle damage, as well as reductions in muscular performance, occur following strenuous activity specific to the occupation of firefighting. This information is particularly relevant, as it implies a firefighter’s capability to perform routine tasks may be impaired during shifts in which prolonged, or repetitively strenuous tasks, must be performed. The results of this study also suggest that if our intervention did replicate the workload of a typical shift, the model of a 24 h shift followed by 48 h of rest is appropriate for recovery.

The strengths of this include a novel insight into the simulated firefighter tasks and provide unique information on occupational health among firefighters. Future studies may expand upon this topic by exploring this relationship, while also assessing muscular performance, in response to multiple bouts of activity (e.g., repetitive PAT attempts). Moreover, it would seem prudent to further determine if there are any associations between markers of inflammation and muscle damage, as well as muscular performance, on PAT performance (i.e., time trial or outcomes specific to the PAT), while also assessing any potential effects of age, experience in the profession, obesity, and training status. A possible explanation for the decrease in physical performance without an elevation in markers of muscle damage and inflammation may be related to neuromuscular fatigue. The use of electromyography to assess maximal voluntary neural activation may be useful in future studies utilizing a similar protocol.

## Figures and Tables

**Table 1:** Participant Characteristics (*n* = 24).

	Mean ± SD
Age (years)	31.2 ± 4.3
Height (cm)	178.9 ± 5.7
Body Mass (kg)	94.2 ± 8.9
BMI (kg/m^2^)	29.5 ± 2.9
PAT Performance (min)	31.2 ± 6.1

Note. BMI = body mass index, calculated as body mass (kg)/height (m^2^); PAT = physical abilities test.

**Table 2:** Dynamic Warm-Up.

Exercise	Repetitions
Jumping Jacks	20
Knee Pull to Chest	5
Reverse Lunge and Reach	5
Walking Toe Touch	5
Quad Pull and Reach	5
Shin Pull	5
Arm Circles	10
Trunk Rotations	5

**Table 3:** Physical Ability Test.

Event	Description
500 m Row	Row 500 m on a Concept 2 rower on resistance setting 5 out of 10. A maximum of 4 minutes permitted.
Ladder Climb	Wearing the proper PPE, climb a 75′ aerial ladder, fully extended, at a 72-degree angle and return to the starting position on the pedestal. There is a 5-min. time maximum. Each rung touched in the assent and decent.
Hose Drag	Drag 45.7 m of charged hose 30.5 m. Pull 7.6 m of the charged hose around at 90°. If fail to properly complete the task, complete the task before moving on.
Stair Climb with Hose Pack	Climb the stairs while carrying the hose pack. If fail to properly complete the task, complete the task before moving on. The hose must be carried, not dragged or tossed. If it is, return to the point the improper action took place and continue. After reaching the third floor, place the hose pack in the red square before moving on to the next step. Skipping steps are not allowed while climbing the stairs.
Hose Pull	Pull two sections of a hose up via the provided rope one at a time. The hand over hand method is to be used. Pulling the rope over the railing to utilize the railing like a pulley is not allowed. The rope may rest on the railing if needed. If the railing is utilized as a pulley, the advantage gained will be eliminated by stopping and the hose is required to be lowered to the position where the advantage began. Begin to raise the hose again.
Crawl	Starting at the designated cone, crawl on hands and knees 19.5 m.
Stair Descent	Carry the hose pack downstairs. The hose must be carried, not dragged or tossed. If it is, return to the point the improper action took place and continue. After reaching the first floor, place the hose pack in the red square before moving on to the next step. Skipping steps is not allowed on decent of the stairs.
Kaiser Machine	Utilizing the provided sledge, strike the weight with the sledge to move the weight the prescribed distance. Hands must rise to head-level on the up stroke.
Hose Couple	The task must be finished and performed properly before moving on. Two sections of 0.9 m hose will be stretched out 0.9 m apart with a nozzle at the end of the hose 0.9 m away. Pull the first section of hose to the second section of hose and couple them together. Pull both sections to the nozzle and couple the nozzle to the hose.
Dummy Drag	Move the dummy the prescribed distance (12.2 m), utilizing any carry desired.

## Data Availability

Not applicable.
